# Prevalence, antimicrobial resistance, and extended-spectrum β-lactamase gene profiles of *Escherichia coli* isolated from poultry eggshells in Can Tho City, Vietnam

**DOI:** 10.14202/vetworld.2025.3640-3650

**Published:** 2025-11-29

**Authors:** Nguyen Thi Lien, Thach Thi Si Huyen, Quach Nguyen Thuy Anh, Tran Minh Dat, Ngo Khanh Duy, Nguyen Tang Phu, Tran Thi Thanh Khuong

**Affiliations:** 1 Department of Microbial Biotechnology, Institute of Food and Biotechnology, Can Tho University, Can Tho City, Vietnam; 2 Department of Molecular Biology, Institute of Food and Biotechnology, Can Tho University, Can Tho City, Vietnam

**Keywords:** *bla*_CTX-M_, Eggshell contamination, *Escherichia coli*, extended-spectrum β-lactamase, multidrug resistance, One Health, Vietnam

## Abstract

**Background and Aim:**

The emergence of extended-spectrum β-lactamase (ESBL)-producing *Escherichia coli* in food sources poses a growing threat to public health. Poultry eggs may act as vehicles for these resistant bacteria, facilitating their transmission through the food chain. This study aimed to determine the prevalence, antimicrobial resistance (AMR) patterns, and ESBL-encoding genes among *E. coli* isolates recovered from chicken, duck, and quail eggs sold in Can Tho City, Vietnam.

**Materials and Methods:**

A total of 900 eggs (300 each from chicken, duck, and quail) were collected from local markets, retail stores, and supermarkets between June and December 2024. *E. coli* isolates were identified through biochemical tests and polymerase chain reaction (PCR) targeting the *uidA* gene. ESBL production was determined using the disk diffusion method, and the presence of *bla*_CTX-M_, *bla*_TEM_, *bla*_NDM_, and *bla*_SHV_ genes was confirmed by PCR. Antimicrobial susceptibility was tested against 10 antibiotics representing eight classes following Clinical and Laboratory Standards Institute (2021) guidelines.

**Results:**

Out of 179 pooled samples positive for *E. coli*, 52 isolates (29.1%) were confirmed as ESBL producers. The highest prevalence was observed in chicken (32.4%) and duck (32.8%) eggs, while quail eggs showed a prevalence of 20%. ESBL-producing isolates most frequently carried *bla*_CTX-M_ (65.4%) and *bla*_TEM_ (44.2%) genes, whereas *bla*_NDM_ was detected in 1.9% of isolates and *bla*_SHV_ was absent. All isolates were multidrug-resistant, exhibiting resistance to 4–9 antibiotic classes. High resistance was observed to amoxicillin-clavulanate (69.2%), ceftriaxone (69.2%), tetracycline (75%), and trimethoprim-sulfamethoxazole (61.5%). No *E. coli* was detected in supermarket eggs, suggesting improved hygiene practices reduce contamination.

**Conclusion:**

The detection of ESBL-producing and multidrug-resistant *E. coli* on poultry eggshells underscores a significant public health concern. The predominance of *bla*_CTX-M_ and *bla*_TEM_ genes highlights the risk of resistance gene dissemination through the egg supply chain. Enhanced surveillance, responsible antibiotic use, and strict hygiene control in small-scale poultry production systems are urgently needed to mitigate the spread of AMR under the One Health framework.

## INTRODUCTION

The administration of antibiotics in food-producing animals has long been a widespread practice aimed at preventing disease outbreaks and improving growth performance and productivity [1]. However, the excessive and often indiscriminate use of antimicrobials has hastened the emergence and dissemination of antimicrobial resistance (AMR), now recognized as a critical global public health and One Health issue [2]. Resistant bacteria and their associated resistance genes can spread along multiple pathways, including the food chain, direct contact between animals and humans, and environmental routes such as contaminated waste, soil, or bio-aerosols [3]. This multifaceted transmission presents serious challenges to animal health, food safety, and human medicine.

Among the various mechanisms of antibiotic resistance, the production of extended-spectrum β-lactamases (ESBLs) is of particular concern. ESBLs are enzymes predominantly produced by Gram-negative bacteria, conferring resistance to a broad spectrum of β-lactam antibiotics, including penicillins, monobactams, and first- to third-generation cephalosporins [4]. Infections caused by ESBL-producing bacteria are associated with limited treatment options, prolonged hospitalization or therapy, increased mortality, and higher healthcare and production costs [5].

Poultry farming represents one of the major livestock sectors in Vietnam, encompassing chickens, ducks, perching ducks, geese, and quails raised for both meat and egg production [6]. Eggs are a vital and affordable protein source, valued for their high nutritional content and accessibility [7]. However, eggs can act as carriers for foodborne pathogens such as *Escherichia coli*, *Enterococci*, *Staphylococcus aureus* [8], and *Salmonella* spp. [9]. Among these, *E. coli* is a major pathogen responsible for avian colibacillosis, which leads to substantial morbidity and mortality in poultry populations [10]. The bacterium can colonize the reproductive tract of infected hens, contaminating the egg yolk and albumen and thereby enabling potential transmission to humans through egg consumption [11].

Although the global prevalence of ESBL-producing *E. coli* in poultry has been increasingly reported [12–14], data on the occurrence of these resistant strains in eggs remain scarce, particularly in developing countries where biosecurity measures and surveillance systems are often insufficient. In Vietnam, the poultry industry produced approximately 15.1 billion eggs in 2020 [6].

Despite growing global concern about AMR in foodborne bacteria, most studies on ESBL-producing *E. coli* have focused on isolates from poultry meat, feces, and farm environments rather than eggs. Limited attention has been given to eggshells, which represent a critical interface between the poultry production environment and human consumers. In Vietnam and other developing countries, the majority of table eggs are distributed through informal markets and smallholder farms where hygienic practices, biosecurity, and antibiotic-use monitoring are insufficiently regulated. These conditions may facilitate bacterial contamination and the persistence of multidrug-resistant strains on egg surfaces. However, there is currently a lack of comprehensive data on the prevalence, genetic determinants, and AMR profiles of ESBL-producing *E. coli* associated with poultry eggs in the Mekong Delta region.

Previous studies [15–17] have mainly documented *E. coli* contamination rates or general antibiotic resistance patterns in poultry, but have rarely combined phenotypic screening for ESBL production with molecular detection of resistance genes such as *bla*_CTX-M_, *bla*_TEM_, *bla*_NDM_, and *bla*_SHV_. Moreover, comparisons among egg types (chicken, duck, and quail) and their respective marketing sources (local markets, retail stores, and supermarkets) are lacking. This knowledge gap limits understanding of the potential public health risks and transmission dynamics of ESBL-producing *E. coli* through egg supply chains in Vietnam’s rapidly expanding poultry industry.

This study aimed to address the aforementioned knowledge gaps by investigating the occurrence and characteristics of ESBL-producing *E. coli* isolated from chicken, duck, and quail eggs collected from various retail sources in Can Tho City, Vietnam. Specifically, the objectives were to:

1. Determine the prevalence of *E. coli* and ESBL-producing *E. coli* on the surface of poultry eggs;
2. Identify the presence of ESBL-encoding genes (*bla*_CTX-M_, *bla*_TEM_, *bla*_NDM_, and *bla*_SHV_) among the isolates using polymerase chain reaction (PCR); and
3. Assess antimicrobial susceptibility patterns of ESBL-producing isolates against commonly used antibiotic classes.

By integrating phenotypic and genotypic analyses, this research aims to provide critical baseline data on AMR dissemination through poultry eggs in Vietnam. The findings are expected to support evidence-based interventions for antibiotic stewardship, biosecurity enhancement, and risk mitigation under a One Health framework linking animal, food, and human health sectors.

## MATERIALS AND METHODS

### Ethical approval

This study did not involve live animals, animal handling, invasive procedures, or human participants. All samples were collected exclusively from the *external surfaces of commercially available eggs* purchased from local markets, retail stores, and supermarkets. As eggshell swabbing does not fall under activities requiring ethical clearance according to Vietnamese regulations and international guidelines for research involving animals, formal approval from an Institutional Animal Care and Use Committee or equivalent body was not required.

All laboratory procedures, including bacterial isolation, phenotypic ESBL screening, PCR assays, and antimicrobial susceptibility testing, were conducted in compliance with Biosafety Level 2 standards as recommended by the Centers for Disease Control and Prevention and the National Institutes of Health (BMBL 6^th^ Edition, 2020) [18]. Standard aseptic practices, appropriate PPE, and safe waste-disposal protocols were strictly followed throughout the study.

### Study period and location

The study was conducted from June 2024 to February 2025 at the Microbiology Laboratory, Institute of Food and Biotechnology, Can Tho University, Can Tho City, Vietnam.

### Sample collection and handling

A total of 900 eggs (300 each from chicken, duck, and quail) with intact shells and no visible cracks were collected from various retail sources, including local markets (10°02’59.0”N 105°46’22.6”E; 10°00’22.7”N 105°45’03.5”E; 10°03’46.7”N 105°45’15.3”E), retail stores (9°59’51.4”N 105°40’09.4”E; 10°01’48.1”N 105°46’08.6”E; 10°01’55.3”N 105°45’47.7”E), and supermarkets (10°00’13.3”N 105°45’10.2”E; 10°02’52.7”N 105°46’47.0”E; 9°59’51.8”N 105°40’09.1”E; 10°01’20.4”N 105°48’00.2”E) in Can Tho City, Vietnam.

Sampling was carried out from June to December 2024, coinciding with the rainy season in southern Vietnam. The sites were geographically distinct to ensure representative coverage of the city’s retail network.

Eggs were transported in sterile polypropylene bags within insulated foam boxes lined with shock-absorbing padding to prevent cracking. Samples were grouped by seller and batch characteristics such as collection time, source, and physical appearance. Eggs from markets and small stores primarily originated from household or smallholder farms with limited traceability, whereas supermarket eggs were supplied by commercial production companies. To ensure adequate representation and manageable processing, three eggs from each batch were pooled as one composite sample.

### Isolation and identification of *E. coli*

Egg surfaces were swabbed aseptically using sterile cotton swabs moistened with 0.85% sterile sodium chloride (NaCl) solution. Each swab tip was transferred into a tube containing 1 mL sterile saline and vortexed gently. Serial dilutions (10¹–10³) were prepared, and 100 μL aliquots were spread on eosin methylene blue agar (Condalab, Spain), followed by incubation at 35°C for 24 h.

Characteristic, metallic-green or black-centered colonies (three per pooled sample) were subcultured on MacConkey agar (Condalab, Spain) and incubated at 35°C for an additional 24 h. Pink colonies on MacConkey agar were subjected to standard biochemical tests, including Gram staining, methyl red, indole, Voges–Proskauer (VP), and citrate utilization (API 20E, bioMérieux) [19].

Isolates consistent with *E. coli* (Gram-negative, rod-shaped, lactose-fermenting, methyl red positive, VP negative, and citrate negative) were retained. To prevent redundancy, one representative isolate per pooled sample was selected for further analyses.

### Molecular confirmation of *E. coli*

Genomic confirmation of *E. coli* was performed using PCR targeting the *uidA* gene as described by Titilawo *et al*. [20]. DNA was extracted from 24-h cultures using the method described by Chen and Kuo [21], and the concentration and purity of the extracted DNA were verified using a NanoDrop 1000 spectrophotometer (Thermo Fisher, USA).

PCR amplification was conducted in a 25 μL reaction mixture containing 1× Taq DNA polymerase buffer (Bioline, UK), 0.4 μM of each primer (Phu Sa Genomics, Vietnam), 1.25 U Taq DNA polymerase (Bioline), and 2 μL of DNA template. Thermocycling conditions were as follows: initial denaturation at 94°C for 5 min; 35 cycles of 94°C for 30 s, 58°C for 1 min, and 72°C for 1 min; and a final extension at 72°C for 8 min.

PCR products were resolved on 1.5% agarose gel (BioBasic, Canada) containing SafeView Classic (ABM, Canada) and visualized using a Bio-Rad UV 2000 gel documentation system. The expected amplicon size for the *uidA* gene was 147 bp. *E. coli* ATCC 25922 served as the positive control.

### Phenotypic detection of ESBL-producing *E. coli*

ESBL production was determined using the disk diffusion method according to Clinical and Laboratory Standards Institute (CLSI) guidelines [22]. *E. coli* isolates were cultured on Luria–Bertani agar for 24 h, and suspensions were adjusted to a 0.5 McFarland standard using 0.85% sterile NaCl. A 0.1 mL inoculum was spread on Mueller–Hinton agar plates (Condalab, Spain).

Antibiotic discs tested included cefotaxime (30 μg), cefotaxime + clavulanate (30/10 μg), ceftazidime (30 μg), and ceftazidime + clavulanate (30/10 μg) (Sigma-Aldrich, Germany). Plates were incubated at 35°C for 18–20 h, and inhibition zones were measured using a digital caliper. An increase of ≥5 mm in the inhibition zone for antibiotic-clavulanate combinations compared to the antibiotic alone was interpreted as ESBL positive. All tests were performed in duplicate.

### Molecular detection of ESBL-encoding genes

Genomic DNA from confirmed ESBL-producing isolates was used for PCR detection of ESBL-encoding genes (*bla*_CTX-M_, *bla*_TEM_, *bla*_NDM_, and *bla*_SHV_) (Table 1) [20, 23–26]. DNA extraction was performed using Chen and Kuo’s method [21]. PCR was conducted in 25 μL reaction mixtures containing double-distilled water, 1× MyTaq buffer (Bioline), 0.4 μM of each primer, 1.25 U MyTaq DNA polymerase, and 2 μL of template DNA.

**Table 1:** Primer sequences used for detecting *uidA* and ESBL-encoding genes in *Escherichia coli* isolates.

Target gene	Sequence (5’- 3’)	Primer annealing temperature	Product size (bp)	Reference
*uidA*	AAAACGGCAAGAAAAAGCAG ACGCGTGGTTAACAGTCTTGCG	58	147	[20]
*bla*_CTX-M_	ATGTTGCAGYACCAGTAARGTKATGGC	55	592	[23]
	TGGGTRAARTARGTSACCAGAAYSAGCGG			
*bla*_TEM_	CATTTCCGTGGTCGCCCTTAT	55	793	[24]
	TCCATAGTTGCCTGACTCCC			
*bla*_SHV_	TCGCCTGTGTATTATCTCCC	58	768	[25]
	CGCAGATAAATCACCACAATG			
*bla*_NDM_	GGTTTGGCGATCTGGTTTTC	52	621	[26]
	CGGAATGGCTCATCACGATC			

Thermocycling conditions were as follows: initial denaturation at 94°C for 3 min; 35 cycles of 94°C for 1 min, annealing at 52°C (*bla*_NDM_), 55°C (*bla*_CTX-M_ and *bla*_TEM_), or 58°C (*bla*_SHV_) for 1 min; and extension at 72°C for 1 min, followed by a final extension at 72°C for 10 min.

PCR products were separated by electrophoresis on 1.5% agarose gels and visualized under UV light. A 100 bp DNA ladder (Bioline) was used for size estimation. *C. freundii* CT1.1 (carrying *bla*_CTX-M_ and *bla*_TEM_) and *K. quasipneumoniae* PG1.3 (carrying *bla*_NDM_ and *bla*_SHV_) served as positive controls. The presence of amplicons at expected sizes was interpreted as positive for the corresponding genes. Each test was performed twice to ensure reproducibility.

### Antimicrobial susceptibility testing

All ESBL-producing *E. coli* isolates were tested for susceptibility to ten antimicrobial agents representing eight antibiotic classes, following CLSI (2021) guidelines [22]. The antibiotics tested were as follows:

- β-lactams: Amoxicillin-clavulanate (AMC, 20/10 μg) and ampicillin (AMP, 10 μg).
- Cephalosporins: Cefoxitin (FOX, 30 μg), ceftriaxone (CRO, 30 μg), and cefuroxime (CXM, 30 μg).
- Quinolone: Ciprofloxacin (CIP, 5 μg).
- Tetracycline: Tetracycline (TET, 30 μg).
- Folate pathway inhibitor: Trimethoprim/sulfamethoxazole (SXT, 1.25/23.75 μg).
- Aminoglycoside: Gentamicin (GEN, 10 μg).

Bacterial suspensions were standardized to 0.5 McFarland, and 0.1 mL aliquots were spread on Mueller–Hinton agar. Antibiotic discs (6 mm) were applied and plates incubated at 35°C for 18–20 h. Inhibition zones were measured using a digital caliper, and results were interpreted as resistant, intermediate, or susceptible according to CLSI breakpoints. *E. coli* ATCC 25922 was used as a control strain.

### Colistin susceptibility testing

Given the continued use of colistin in Vietnam’s poultry production, all ESBL-producing isolates were also tested for colistin resistance using the microbroth dilution method with colistin sulfate (COL) (Sigma-Aldrich, Germany) following CLSI (2021) standards [22]. All assays were performed in triplicate.

### Statistical analysis

The prevalence of *E. coli* and ESBL-producing *E. coli*, as well as the distribution of ESBL-encoding genes and AMR profiles across egg types, were expressed as frequencies and percentages. Differences among chicken, duck, and quail eggs were analyzed using the Chi-square goodness-of-fit test in Minitab version 16 (Minitab Inc., USA). p < 0.05 was considered statistically significant.

## RESULTS

### Prevalence and identification of *E. coli* isolates

Out of 300 samples collected from chicken, duck, and quail eggs, 179 were positive for *E. coli*. All 179 isolates exhibited typical phenotypic characteristics consistent with those described by Feng *et al*. [19] and were confirmed as *E. coli* through PCR amplification of a 147-bp fragment of the *uidA* gene [20]. Among the total isolates, 68 (38.0%), 61 (34.1%), and 50 (27.9%) originated from chicken, duck, and quail eggs, respectively. Most *E. coli* isolates were recovered from eggs obtained at local markets and retail stores, while no *E. coli* was detected in any samples collected from supermarkets. The absence of contamination in supermarket eggs can be attributed to the surface cleaning and packaging practices implemented by large-scale commercial producers, which minimize direct human contact and environmental exposure during handling and display.

### Prevalence of ESBL-producing *E. coli*

All 179 *E. coli* isolates were screened phenotypically for ESBL production using the disk diffusion method. Of these, 52 isolates (29.1%) were confirmed as ESBL producers.

The prevalence of ESBL-producing *E. coli* among chicken, duck, and quail eggs was 32.4%, 32.9%, and 20.0%, respectively. Although chicken and duck eggs showed higher proportions of ESBL-positive isolates compared to quail eggs, the differences were not statistically significant (p = 0.155) (Table 2).

**Table 2:** Prevalence of *E. coli* and ESBL-producing *E. coli* isolated from the surface of chicken, duck, and quail eggs collected in Can Tho City, Vietnam.

*E. coli* categories	Chicken (n = 68) n (%)	Duck (n = 61) n (%)	Quail (n = 50) n (%)	Total (n = 179) n (%)	p-value
*E. coli*	46 (67.6)	41 (67.2)	40 (80)	127 (70.9)	0.477
ESBL-producing *E. coli*	22 (32.4)	20 (32.8)	10 (20)	52 (29.1)	0.155

*E. coli* = E*scherichia coli*, ESBL = Extended-spectrum β-lactamase.

### Distribution of ESBL-encoding genes

Among the 52 ESBL-producing isolates, 48 (92.3%) harbored at least one ESBL-encoding gene, including *bla*_CTX-M_, *bla*_TEM_, or *bla*_NDM_, while none carried *bla*_SHV_.

The *bla*_CTX-M_ gene was the most frequently detected, particularly in isolates from chicken eggs (78.9%), followed by those from duck eggs (52.6%) (p < 0.001). The *bla*_TEM_ gene was predominant among ESBL-producing *E. coli* isolates from quail eggs (90.0%), whereas *bla*_NDM_ was detected in a single isolate (1.9%).

The most common gene combination was *bla*_CTX-M_ + *bla*_TEM_, observed in isolates from duck (25.3%) and chicken (15.8%) eggs. The simultaneous presence of three genes (*bla*_CTX-M_, *bla*_TEM_, and *bla*_NDM_) was detected in only one quail egg isolate. A summary of the gene distribution is provided in Table 3.

**Table 3:** Distribution of ESBL-encoding genes among ESBL-producing *Escherichia coli* isolates isolated from chicken, duck, and quail eggs.

ESBL-encoding gene profiles	Chicken (n = 19) n (%)	Duck (n = 19) n (%)	Quail (n = 10) n (%)	Total (n = 48) n (%)	p-value
*bla*_CTX-M_	15 (78.9)	10 (52.6)	0 (0)	25 (52.1)	<0.001
*bla*_TEM_	1 (5.3)	4 (21.1)	9 (90)	14 (29.2)	<0.001
*bla*_CTX-M_ + *bla*_TEM_	3 (15.8)	5 (25.3)	0 (0)	8 (16.7)	<0.001
*bla*_CTX-M_ + *bla*_TEM_ + *bla*_NDM_	0	0	1 (10)	1 (2.1)	<0.001

ESBL = Extended-spectrum b-lactamase.

### AMR profiles of ESBL-producing *E. coli*

ESBL-producing *E. coli* isolates demonstrated high resistance to multiple antibiotic classes. The highest resistance rates were observed for AMP (98.1%), cephalosporins (FOX: 88.5%; CXM: 92.3%; and CRO: 69.2%), and β-lactam/β-lactamase inhibitor combinations (AMC: 69.2%).

High resistance frequencies were also recorded for TET (75%), SXT (61.5%), and colistin (94.2%). In contrast, lower resistance rates were noted for CIP (23.1%) and GEN (32.7%).

Notably, isolates from chicken eggs showed significantly higher resistance to AMC, CRO, TET, and SXT compared with those from duck and quail eggs (p < 0.05). Conversely, quail egg isolates exhibited significantly higher resistance to CIP and GEN than isolates from the other two poultry species (p < 0.05) (Table 4).

**Table 4:** Antibiotic resistance profiles of ESBL-producing *Escherichia coli* isolates recovered from chicken, duck, and quail eggs.

Antimicrobial agents	Chicken (n = 22) n (%)	Duck (n = 20) n (%)	Quail (n = 10) n (%)	Total (n = 48) n (%)	p-value
Amoxicillin-clavulanate	20 (90.9)	9 (45)	7 (70)	36 (69.2)	<0.001
Ampicillin	21 (95.5)	20 (100)	10 (100)	51 (98.1)	0.934
Cefoxitin	17 (77.3)	20 (100)	9 (90)	46 (88.5)	0.234
Ceftriaxone	16 (72.7)	6 (30)	4 (40)	36 (69.2)	<0.001
Cefuroxime	18 (81.8)	20 (100)	10 (100)	48 (92.3)	0.309
Ciprofloxacin	3 (13.6)	5 (25)	4 (40)	12 (23.1)	<0.001
Colistin	19 (86.4)	20 (100)	10 (100)	49 (94.2)	0.524
Gentamicin	5 (22.7)	3 (15)	9 (90)	17 (32.7)	<0.001
Tetracycline	21 (95.5)	12 (60)	6 (60)	39 (75)	0.003
Trimethoprim/sulfamethoxazole	17 (77.3)	13 (65)	2 (20)	32 (61.5)	<0.001

ESBL = Extended-spectrum b-lactamase.

### Multidrug resistance (MDR) patterns

All 52 ESBL-producing *E. coli* isolates were resistant to between four and nine antibiotics, classifying them as MDR. A total of 31 distinct MDR profiles were identified.

The most frequent resistance pattern involved seven antibiotics, followed by those involving eight antibiotics. The number of unique MDR patterns recorded among isolates from chicken, duck, and quail eggs was 16, 13, and 7, respectively.

Among chicken isolates, the most common MDR profile was AMC–AMP–FOX–CRO–TET–SXT–CXM–COL (18.2%), followed by AMC–AMP–FOX–TET–SXT–CXM–COL (9.1%). In duck isolates, the predominant pattern was AMP–FOX–SXT–CXM–COL (20%), whereas quail isolates most frequently exhibited the AMC–AMP–FOX–TET–GEN–CXM–COL pattern (40%) (Table 5).

**Table 5:** Multidrug resistance profiles of ESBL-producing *Escherichia coli* isolates recovered from chicken, duck, and quail eggs.

MDR pattern	ESBL-producing isolates			

	Chicken (n = 22)	Duck (n = 20)	Quail (n = 10)	Total (n = 52)
AMC-AMP-FOX-CRO-TET-SXT-CXM-COL	4 (18.2)	1 (5)	0	5 (9.6)
AMC-AMP-FOX-TET-SXT-CXM-COL	2 (9.1)	3 (15)	0	5 (9.6)
AMC-AMP-FOX-TET-GEN-CXM-COL	0	0	4 (40)	4 (7.7)
AMP-FOX-SXT-CXM-COL	0	4 (20)	0	4 (7.7)
AMC-AMP-FOX-TET-CXM-COL	1 (4.5)	2 (10)	0	3 (5.8)
AMC-AMP-FOX-CRO-CIP-TET-SXT-CXM-COL	2 (9.1)	0	0	2 (3.8)
AMC-AMP-FOX-CIP-TET-GEN-CXM-COL	0	1 (5)	1 (10)	2 (3.8)
AMC-AMP-FOX-CRO-TET-CXM-COL	2 (9.1)	0	0	2 (3.8)
AMP-FOX-CRO-CIP-SXT-CXM-COL	0	1 (5)	1 (10)	2 (3.8)
AMP-FOX-CXM-COL	0	2 (10)	0	2 (3.8)
Other (≤1 isolate/pattern)	11 (50)	6 (30)	4 (40)	21 (40.4)

MDR = Multidrug resistance, ESBL = Extended-spectrum b-lactamase, AMC = Amoxicillin-clavulanate, AMP = Ampicillin, FOX = Cefoxitin, CRO = Ceftriaxone, CXM = Cefuroxime, CIP = Ciprofloxacin, TET = Tetracycline, SXT = Trimethoprim/sulfamethoxazole, GEN = Gentamicin, COL = Colistin sulfate.

### Summary of findings

**Figure 1:**
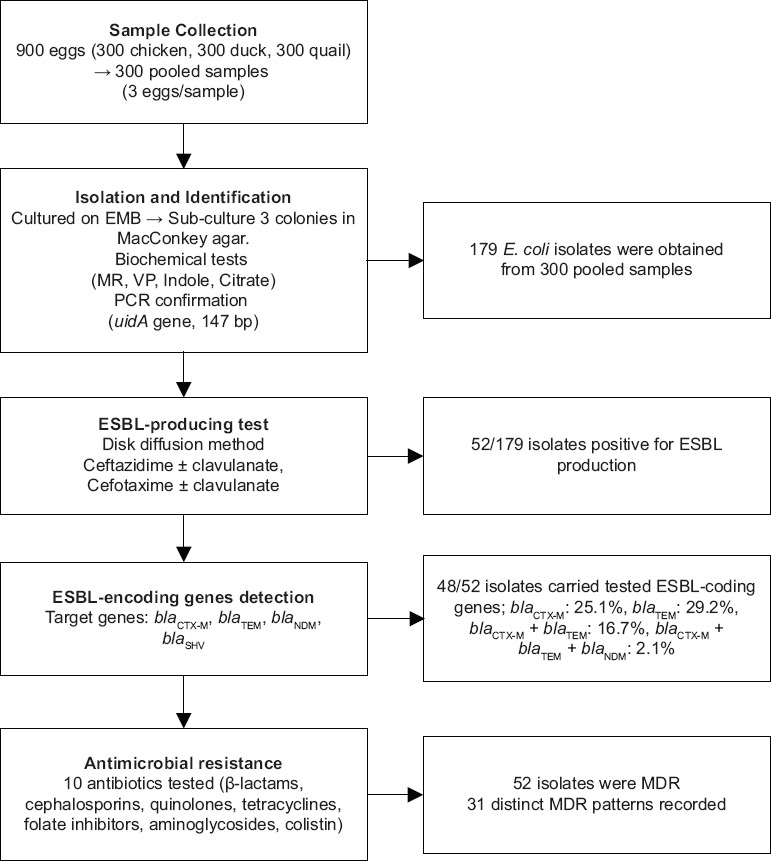
Flow chart illustrating the detection and characterization of extended-spectrum β-lactamase-producing *Escherichia coli* isolated from poultry eggshells.

The results reveal a high prevalence of *E. coli* contamination in poultry eggs sourced from traditional markets and small-scale retailers, contrasted by the absence of contamination in supermarket eggs. The detection of multiple ESBL genes, particularly *bla*_CTX-M_ and *bla*_TEM_, and the universal presence of MDR highlight the potential role of poultry eggs as reservoirs and transmission vehicles for antimicrobial-resistant bacteria in Vietnam’s retail food chain (Figure 1).

## DISCUSSION

### Prevalence of *E. coli* contamination on eggshells

*E. coli* is a commensal bacterium in the intestinal flora of animals and humans; however, certain strains can cause severe infections in humans, mammals, and birds [27]. In the present study, *E. coli* was detected in 179 out of 300 egg samples (59.7%), with the majority (74.6%) of positive isolates obtained from eggs sold at local markets and retail stores. Notably, no *E. coli* was detected in eggs collected from supermarkets.

This finding aligns with results from Bangladesh (80%) [28] and Pakistan (85.4%) [29], but the prevalence was higher than that reported in Hanoi, Vietnam (29.1% in chicken and 30% in duck eggs) [17], and Thailand (12.1%) [30]. Variations in *E. coli* contamination across studies may be attributed to differences in farm hygiene, biosecurity practices, storage conditions, and egg handling during marketing and transportation.

### Influence of farm type and egg handling practices

In Vietnam, eggs from smallholder and backyard farms constitute a major portion of the national supply [6]. These farms typically operate under non-intensive systems with limited biosecurity, leading to higher microbial contamination risks. Eggs produced by such systems are often unwashed and distributed through informal market channels [6], making them more susceptible to contamination from environmental sources.

A study by Hsu *et al*. [31] in Taiwan also reported the presence of *Staphylococcus*, *Enterobacteriaceae*, and *E. coli* in unwashed eggs, which supports the current findings. In contrast, eggs from supermarkets in Vietnam are mainly produced by large-scale companies that apply cleaning, grading, and packaging procedures before retail distribution [32]. The absence of *E. coli* in supermarket samples emphasizes that hygienic processing and packaging substantially reduce microbial contamination and improve food safety.

### Study limitations and implications

The present study provides valuable baseline data on *E. coli* contamination in eggs from chickens, ducks, and quails in Can Tho City. However, some limitations exist, including the use of pooled samples and the limited geographic coverage of the study area. In addition, the potential co-contamination of eggshells with other pathogens warrants further investigation. Despite these constraints, the findings underscore the urgent need for improved hygiene management, sanitation, and microbiological monitoring in informal poultry production and retail systems to reduce pathogen transmission to consumers.

### Prevalence and distribution of ESBL-producing *E. coli*

The ability of pathogens to produce ESBLs significantly compromises the efficacy of antibiotics and represents a major public health threat. In this study, 29.1% of *E. coli* isolates were confirmed as ESBL producers. The *bla*_CTX-M_ gene was detected in 81.8% and 75% of ESBL-producing isolates from chicken and duck eggs, respectively, while all isolates from quail eggs carried *bla*_TEM_, and one isolate harbored *bla*_NDM_.

The prevalence of ESBL-producing *E. coli* from eggs in this study was lower than that reported from boot or cloacal swabs [33, 34], but higher than findings based on meat, viscera [35], or cecal samples [13]. The detection of *bla*_CTX-M_ and *bla*_TEM_ genes aligns with previous reports, indicating their widespread presence in poultry-associated *E. coli*. The absence of *bla*_SHV_ suggests potential influence of sample type and geographical variation on ESBL gene profiles.

The detection of *bla*_NDM_, a carbapenemase-encoding gene, in one isolate is particularly concerning. This highlights the potential for horizontal transmission of highly resistant determinants within the food chain and emphasizes the need for ongoing genomic surveillance of foodborne *E. coli* isolates.

### Possible sources and transmission of ESBL genes

Eggshell contamination can occur through multiple routes, including contact with feces, dust, soil, or contaminated washing water during collection, transport, and storage [12]. Among ESBLs, CTX-M-type enzymes are the most clinically relevant and are often detected in poultry-associated *E. coli* isolates worldwide [4].

The distribution of ESBL genes exhibits considerable regional variation due to differences in antibiotic usage and farm management. These genes are often plasmid-mediated and associated with mobile genetic elements such as transposons and insertion sequences [36], facilitating their horizontal transfer through transformation, transduction, or conjugation [37]. The results of this study indicate that poultry eggs may act as vehicles for ESBL-producing *E. coli*, potentially transferring resistant bacteria from farm environments to consumers. Future research employing whole-genome sequencing or multilocus sequence typing would be valuable for elucidating clonal relationships and transmission dynamics among isolates.

### AMR and MDR patterns

The ESBL-producing *E. coli* isolates in this study exhibited extensive resistance to multiple antibiotic classes, including β-lactams, cephalosporins, sulfonamides, TETs, and polymyxins. Thirty-one distinct MDR patterns were identified, underscoring the complexity of AMR dissemination.

Overall resistance rates were higher than those reported for *E. coli* from poultry eggs in Hanoi (65.2%) [17] and Thailand (57.1%) [30] and are consistent with the global meta-analysis reporting elevated resistance in table eggs [38]. These findings reinforce the critical need for antibiotic stewardship in poultry production systems.

### Drivers of AMR

The widespread use of antibiotics at sub-therapeutic doses for growth promotion, prophylaxis, and treatment in poultry farming has been a major driver of AMR emergence [39]. Many antimicrobials are poorly absorbed and excreted in feces or urine, leading to environmental contamination [40]. These residues exert selective pressure on bacterial populations, promoting the evolution of resistant strains [3].

Furthermore, the highly dynamic accessory genome of *E. coli* facilitates horizontal gene transfer, enabling the development of hybrid and MDR populations with enhanced adaptability [41]. This genomic plasticity underscores the difficulty in controlling AMR dissemination in foodborne pathogens.

### Public health and One Health implications

The present study highlights an alarming situation regarding antibiotic resistance in ESBL-producing *E. coli* isolated from poultry eggs in Vietnam. The coexistence of *bla*_CTX-M_, *bla*_TEM_, and *bla*_NDM_ genes, along with MDR, poses a significant One Health concern due to the potential transmission of resistance determinants from poultry to humans through food or the environment.

To mitigate this threat, future research should examine antibiotic usage patterns at the farm level, environmental contamination routes, and the molecular mechanisms underlying resistance. Policymakers should strengthen national regulations on antimicrobial use in layer farms and enforce regular microbiological monitoring of egg products.

Educational programs for poultry farmers should promote responsible antibiotic practices, biosecurity, and proper hygiene during egg handling and collection. Consumers are encouraged to source eggs from certified producers, avoid raw or undercooked eggs, and maintain strict hygiene during food preparation to prevent cross-contamination.

## CONCLUSION

This study provides critical evidence of *E. coli* contamination and the widespread occurrence of ESBL-producing isolates on poultry eggshells in Can Tho City, Vietnam. Among 300 egg samples, 59.7% were positive for *E. coli*, and 29.1% of these isolates exhibited ESBL production. The *bla*_CTX-M_ gene was predominant, followed by *bla*_TEM_ and *bla*_NDM_, while *bla*_SHV_ was absent. All ESBL-producing isolates demonstrated MDR, with high resistance rates to β-lactams, cephalosporins, sulfonamides, and TETs. Chicken and duck eggs had a higher prevalence of ESBL-positive isolates than quail eggs, and no *E. coli* was detected in supermarket eggs, indicating the effectiveness of hygienic production and packaging practices in reducing contamination risk.

The findings emphasize that poultry eggs, especially those sold in traditional markets and small retail stores, can serve as potential vehicles for transmitting antimicrobial-resistant bacteria to humans. Strengthening on-farm biosecurity, implementing egg washing and surface sanitization protocols, and enforcing hygienic packaging standards are essential interventions to mitigate microbial contamination. Moreover, promoting rational antibiotic use in layer production systems and continuous monitoring of AMR patterns should form integral components of national One Health surveillance strategies.

This research represents one of the first integrated assessments in Vietnam that combines phenotypic and genotypic characterization of ESBL-producing *E. coli* from multiple poultry species (chicken, duck, and quail eggs). The study employed validated molecular and antimicrobial susceptibility methods (CLSI 2021) and used internal and external controls to ensure analytical reliability.

The study’s scope was limited by the use of pooled samples and its confinement to a single geographic region, which may not fully represent the national epidemiological situation. In addition, only a limited number of resistance genes were screened, and whole-genome characterization was not performed.

Future studies should extend surveillance to different provinces, incorporate whole-genome sequencing to track mobile genetic elements and clonal lineages, and correlate resistance patterns with antibiotic usage data on farms. Longitudinal studies linking farm environments, egg handling practices, and human exposure pathways would further elucidate the transmission dynamics of ESBL-producing *E. coli* within the poultry value chain.

The detection of *bla*_CTX-M_ - and *bla*_TEM_ -mediated ESBL-producing, MDR *E. coli* on poultry eggshells, highlights a growing public health threat in Vietnam’s egg production sector. These findings underscore the urgent need for a coordinated One Health approach involving policymakers, veterinarians, microbiologists, and farmers to promote responsible antibiotic stewardship, enhance biosafety, and safeguard food safety. Strengthening biosecurity and hygiene from farm to retail level will be crucial to limiting the spread of AMR through poultry products.

## DATA AVAILABILITY

The supplementary data can be made available from the corresponding author upon request.

## AUTHORS’ CONTRIBUTIONS

NTL, NTP, and TTTK: Conceptualization, methodology design, data analysis, supervision, and manuscript drafting and revision. QNTA, NKD, TMD, and TTSH: Sample collection, laboratory experiments, and data curation, PCR validation, antimicrobial susceptibility testing, and statistical analysis. All authors have read and approved the final version of the manuscript.

## References

[1] Selaledi L.A, Hassan Z.M, Manyelo T.G, Mabelebele M (2020). The current status of the alternative use to antibiotics in poultry production:An African perspective. Antibiotics (Basel).

[2] Velazquez-Meza M.E, Galarde-López M, Carrillo-Quiróz B, Alpuche-Aranda C.M (2022). Antimicrobial resistance:One health approach. Vet. World.

[3] Endale H, Mathewos M, Abdeta D (2023). Potential causes of spread of antimicrobial resistance and preventive measures in one health perspective-a review. Infect. Drug Resist.

[4] Widodo A, Khairullah A.R, Effendi M.H, Moses I.B, Agustin A.L.D (2024). Extended-spectrum b-lactamase-producing *Escherichia coli* from poultry:A review. Vet. World.

[5] Maslikowska J.A, Walker S.A, Elligsen M, Mittmann N, Palmay L, Daneman N, Simor A (2016). Impact of infection with extended-spectrum β-lactamase-producing *Escherichia coli* or *Klebsiella* species on outcome and hospitalization costs. J. Hosp. Infect.

[6] Birhanu M.Y, Geremew K, Esatu W, Worku S, Getachew F, Nguyen V.D, Ngo T.K.C, Unger F, Dessie T (2021). Poultry Production, Marketing and Consumption in Vietnam:A Review of Literature. ILRI Research Report 80.

[7] Réhault-Godbert S, Guyot N, Nys Y (2019). The golden egg:Nutritional value, bioactivities, and emerging benefits for human health. Nutrients.

[8] Pesavento G, Calonico C, Runfola M, Nostro A.L (2017). Free-range and organic farming:Eggshell contamination by mesophilic bacteria and unusual pathogens. J. Appl. Poult. Res.

[9] Solís D, Cordero N, Quezada-Reyes M, Escobar-Astete C, Toro M, Navarrete P, Reyes-Jara A (2023). Prevalence of *Salmonella* in eggs from conventional and cage-free egg production systems and the role of consumers in reducing household contamination. Foods.

[10] Azam M, Mohsin M, Sajjad-Ur-Rahman, Saleemi M.K (2019). Virulence-associated genes and antimicrobial resistance among avian pathogenic *Escherichia coli* from colibacillosis affected broilers in Pakistan. Trop. Anim. Health Prod.

[11] Abdelhamid M.K, Hess C, Bilic I, Glösmann M, Rehman H.U, Liebhart D, Hess M, Paudel S (2024). A comprehensive study of colisepticaemia progression in layer chickens applying novel tools elucidates pathogenesis and transmission of *Escherichia coli* into eggs. Sci. Rep.

[12] Blaak H, Van Hoek A.H, Hamidjaja R.A, Van Der Plaats R.Q, Kerkhof-De Heer L, De Roda Husman A.M, Schets F.M (2015). Distribution, numbers, and diversity of ESBL-producing *E. coli* in the poultry farm environment. PloS One.

[13] Sakthikarthikeyan S, Sivakumar M, Manikandan R, Senthilkumar P, Sureshkumar V, Malmarugan S, Prabhu M, Ramakrishnan V (2023). Prevalence and molecular characterization of multidrug-resistant ESBL-producing *E. coli* in commercial poultry. Indian J. Anim. Res.

[14] Aliyu A.B, Jalila A, Saleha A.A, Zunita Z (2024). ESBL producing *E. coli* in chickens and poultry farms environment in Selangor, Malaysia:A cross-sectional study on their occurrence and associated risk factors with environment and public health importance. Zoonoses Public Health.

[15] Thu H.T.V, Anh D.T.L, Dong L.V (2019). *Escherichia coli* infection in ducks in the Mekong Delta:Bacterial isolation, serogroup distribution and antibiotic resistance. Can. Tho. Univ. J. Sci.

[16] Nhung N.T, Yen N.T.P, Dung N.T.T, Nhan N.T.M, Phu D.H, Kiet B.T, Thwaites G, Geskus R.B, Baker S, Carrique-Mas J, Choisy M (2022). Antimicrobial resistance in commensal *Escherichia coli* from humans and chickens in the Mekong Delta of Vietnam is driven by antimicrobial usage and potential cross-species transmission. JAC Antimicrob. Resist.

[17] Thai T.H, Ngan P.H, Huong C.T.H, Ha C.T.H (2017). Antimicrobial resistance of *E. coli* and *Salmonella* isolated from egg at Hanoi City retail markets. Vietnam J. Agri. Sci.

[18] Centers for Disease Control and Prevention National Institutes of Health (2020). Biosafety in Microbiological and Biomedical Baboratories.

[19] Feng P, Weagant D.D, Grant M.A, Burkhardt W (2020). Enumeration of *Escherichia coli* and the Coliform Bacteria. Ch. 4.

[20] Titilawo Y, Obi L, Okoh A (2015). Occurrence of virulence gene signatures associated with diarrhoeagenic and non-diarrhoeagenic pathovars of *Escherichia coli* isolates from some selected rivers in South-Western Nigeria. BMC Microbiol.

[21] Chen W.P, Kuo T.T (1993). A simple and rapid method for the preparation of gram-negative bacterial genomic DNA. Nucleic Acids Res.

[22] Clinical and Laboratory Standards Institute (2021). Performance Standards for Antimicrobial Susceptibility Testing.

[23] Mulvey M.R, Soule G, Boyd D, Demczuk W, Ahmed R, Multi-Provincial *Salmonella* Typhimurium Case Control Study Group (2003). Characterization of the first extended-spectrum β-lactamase-producing *Salmonella* isolate identified in Canada. J. Clin. Microbiol.

[24] Walker R.A, Lindsay E, Woodward M.J, Ward L.R, Threlfall E.J (2001). Variation in clonality and antibiotic-resistance genes among multiresistant *Salmonella enterica* serotype *Typhimurium* phage-type U302 (MR U302) from humans, animals, and foods. Microb. Drug Resist.

[25] Van T.T, Chin J, Chapman T, Tran L.T, Coloe P.J (2008). Safety of raw meat and shellfish in Vietnam:An analysis of *Escherichia coli* isolations for antibiotic resistance and virulence genes. Int. J. Food Microbiol.

[26] Poirel L, Walsh T.R, Cuvillier V, Nordmann P (2011). Multiplex PCR for detection of acquired carbapenemase genes. Diagn. Microbiol. Infect. Dis.

[27] Pokharel P, Dhakal S, Dozois C.M (2023). The diversity of *Escherichia coli* pathotypes and vaccination strategies against this versatile bacterial pathogen. Microorganisms.

[28] Ema F.A, Arif M, Islam M.A, Khatun M.M (2018). Isolation and identification of duck egg-borne bacteria and their antibiogram profile. J. Adv. Vet. Anim. Res.

[29] Ullah R, Afridi M, Ur Rehman M, Noorul Hasan T, Tahir M, Ali Khan H, Shah Z, Ul Ain Q, Perween S, Arshad N, Yousaf M, Ammad M, Hussain A, Hussain S (2023). Antimicrobial resistance in *Escherichia coli* and *Salmonella* spp. isolated from table egg shells and its contents sold at different markets in Peshawar, Pakistan. Microbes Infect. Dis.

[30] Siriphap A, Suwancharoen C, Laenoi W, Kraivuttinun P, Suthienkul O, Prapasawat W (2022). First study on virulence genes, antimicrobial resistance, and integrons in *Escherichia coli* isolated from cage, free-range, and organic commercial eggs in Phayao Province, Thailand. Vet. World.

[31] Hsu S.C, Chen H.L, Chou C.F, Liu W.C, Wu C.T (2023). Characterization of microbial contamination of retail washed and unwashed shell eggs in Taiwan. Food Control.

[32] Vietnam Pictorial (2021). Tien Vien Clean Chicken Eggs.

[33] Gundran R.S, Cardenio P.A, Villanueva M.A, Sison F.B, Benigno C.C, Kreausukon K, Pichpol D, Punyapornwithaya V (2019). Prevalence and distribution of *bla*_CTX-M_, *bla*_SHV_, *bla*_TEM_ genes in extended- spectrum b- lactamase- producing *E. coli* isolates from broiler farms in the Philippines. BMC Vet. Res.

[34] Kendek I.A, Khairullah A.R, Effendi M.H, Wibisono F.J, Tyasningsih W, Ugbo E.N, Budiastuti B, Degu N.Y, Moses I.B, Ahmad R.Z, Yanestria S.M, Kurniasih D.A.A, Raissa R, Rehman S (2025). Detection of the *bla*_TEM_ gene on multidrug-resistant *Escherichia coli* producing extended spectrum b-lactamase from ducks in live poultry markets in Surabaya, Indonesia. Open Vet. J.

[35] El-Tarabili R.M, Hanafy A.S.T, El Feky T.M (2023). Virulence, resistance profile, antimicrobial resistance Genes of *ESBLs, XDR Escherichia coli* isolated from ducks. J. Adv. Vet.

[36] Castanheira M, Simner P.J, Bradford P.A (2021). Extended-spectrum b-lactamases:an update on their characteristics, epidemiology and detection. JAC Antimicrob. Resist.

[37] Husna A, Rahman M.M, Badruzzaman A.T.M, Sikder M.H, Islam M.R, Rahman M.T, Alam J, Ashour H.M (2023). Extended-spectrum b-lactamases (ESBL):Challenges and opportunities. Biomedicines.

[38] Hinson C, Tonouhewa T, Azokpota P, Daube G, Korsak N, Sessou P (2025). Global prevalence and antibiotic resistance profiles of bacterial pathogens in table eggs:A systematic review and meta-analysis. Vet. World.

[39] Ma F, Xu S, Tang Z, Li Z, Zhang L (2021). Use of antimicrobials in food animals and impact of transmission of antimicrobial resistance on humans. Biosaf. Health.

[40] Shao S, Hu Y, Cheng J, Chen Y (2018). Research progress on distribution, migration, transformation of antibiotics and antibiotic resistance genes (*ARGs*) in aquatic environment. Crit. Rev. Biotechnol.

[41] Geurtsen J, De Been M, Weerdenburg E, Zomer A, McNally A, Poolman J (2022). Genomics and pathotypes of the many faces of *Escherichia coli*. FEMS Microbiol. Rev.

